# Expression of a gap junction protein, connexin43, in a large panel of human gliomas: new insights

**DOI:** 10.1002/cam4.730

**Published:** 2016-06-15

**Authors:** Sophie Crespin, Gaëlle Fromont, Michel Wager, Pierre Levillain, Laurent Cronier, Arnaud Monvoisin, Norah Defamie, Marc Mesnil

**Affiliations:** ^1^ Laboratoire Signalisation et Transports Ioniques MembranairesUniversity of PoitiersERL‐CNRS 73681 rue Georges Bonnet, TSA 51106, 86073Poitiers Cedex 09France; ^2^ Service of PathologyPoitiers University HospitalJean Bernard6 rue de La Milétrie, TSA 51115, 86073Poitiers Cedex 09France; ^3^ Service of NeurosurgeryPoitiers University HospitalJean Bernard6 rue de La Milétrie, TSA 51115, 86073Poitiers Cedex 09France

**Keywords:** Brain cancer, connexins, Cx43, gap junctions, glioblastoma, gliomas

## Abstract

Precise diagnosis of low and high grades of brain tumors permits determining therapeutical strategies. So far, diagnosis and prognosis of gliomas were based on histological and genetic criteria which need being completed by a panel of molecular markers. Highly distributed in brain, gap junction proteins, connexins, could be considered as markers of glioma progression as previous studies indicated that expression of a connexin type, connexin43 (Cx43), is inversely correlated to tumor grading. However, this assumption was weakened by the low number of glioma samples used. Taking advantage of tissue microarray technique, we pursued this analysis by studying *in situ* expression of Cx43 on 85 samples (37 grade IV, 18 grade III, 24 grade II, and 6 grades II to III). Our analysis confirmed the global diminution of Cx43 expression in glioblastomas that was observed in previous studies. However, this analysis brought new insights such as the following ones. First, the high number of samples permitted to show that more than 60% of glioblastomas still express Cx43. Second, no gradual decrease in Cx43 expression was observed between grades II and III, but Cx43 appeared to be a marker distinguishing oligodendrocytic and astrocytic grade III tumors. Third, independently from tumor grade, a Cx43 nuclear staining was detected in areas where leukocytes are present. In conclusion, our study emphasizes the importance of *in situ* immunohistochemical approaches by giving more precise insights in the subcellular localization of Cx43. It also emphasizes the necessity to carry out such analysis on a wide range of samples to circumvent the high glioma heterogeneity.

## Introduction

Tumors of nervous system account for less than 2% of malignancies (175,000 cases per year worldwide) and are the 12th most frequent cause of cancer‐related mortality in human 1. In the developed countries, incidence of malignant brain tumors tends to be higher and reaches 6–8 new cases per 100,000 persons per year. Even if low, this incidence is counterbalanced by the fact that the most common brain tumor in adults, glioblastoma, is surgically incurable and resistant to radiotherapy and chemotherapy (only 3% of patients survive more than 3 years). In addition, during past years, incidence of glioblastomas has increased annually by 1–2% without identifying its etiology 2. Because of their rapid evolution and lethality, a precise diagnosis of low and high grades of brain tumors is crucial for determining appropriate therapeutical strategies. So far, their diagnosis mostly refers to the World Health Organization (WHO) classification 2. Based on histological criteria, gliomas are classified into three grades from grade II to grade IV with subtypes depending on their probable cellular origin (astrocytic or oligodendrocytic). Such criteria need to be completed by a panel of molecular markers to state more precisely on diagnosis and prognosis of gliomas, especially for distinguishing low (grade II) and high (grades III and IV) grades.

Highly and specifically distributed in brain, gap junctions could be considered as putative markers of glioma progression. Their structural proteins, connexins (Cx), are members of a multigene family (21 members in human) presenting a specific tissue distribution 3. In brain, 12 members of this multigene family are expressed 4 and some of them are considered as differentiation markers of cell types in which they are expressed (Cx32 in oligodendrocytes, Cx36 in neurons, and Cx43 and Cx30 in astrocytes) 5. Such a specific distribution is related probably to particular cellular functions and predisposes connexins to be abnormally expressed during tumor progression. This assumption is reinforced by the fact that gap junctions mediate gap junctional intercellular communication (GJIC) which favors direct homeostatic diffusion of small hydrophilic molecules (ions, second messengers, metabolites <1200 Da) between cytoplasms of adjacent cells. Tumor growth appearing as homeostasis disruption, the dysfunction of gap junctions leading to low GJIC capacity is a current feature in cancer cells and has been consequently associated with tumor development 6, 7, 8. Reinducing GJIC appeared to have tumor suppressive effects in several cancer cell types 8. And this was also observed in the case of brain tumor cells as transfection of the major astrocytic connexin, Cx43, induces “normalization” of the phenotype (decreased growth) of rat and human glioma cell lines 9, 10. Such data tend to demonstrate that Cx43 is involved in growth regulation and thus, conversely, they suggest that lack of Cx43 expression and/or function might be associated to glioma development and possibly be predictive of its evolution.

Paradoxically, despite of these *in vitro* results, only few attempts have been performed to check Cx43 expression in human gliomas. As expected, these few studies suggest that Cx43 expression is inversely correlated to tumor grade 11, 12, 13, 14. At a first glance, such a conclusion appears to be in accordance with the admitted general assumption that connexin expression and/or function are diminished in tumor cells whatever their tissue origin maybe 6, 7, 8, 15. However, in the brain tumor context, this agreement is weakened by the low number of samples which was analyzed. Moreover, the lack of accuracy about astrocytic or oligodendrocytic origins of the studied tumors prevented to establish Cx43 as a diagnosis or prognosis marker for human gliomas 11, 12, 13.

In order to complete previous studies, and taking advantage of the tissue microarray (TMA) technique, we undertook the analysis of Cx43 expression in human adult gliomas, but on a high number of samples (85 patients). Because of the putative roles of Cx43 in cell proliferation control and invasion, our study focused on its *in situ* expression and localization in selected zones of a wide range of gliomas from grade II to grade IV (24 grade II, 18 grade III, 37 grade IV, and 6 tumors exhibiting mixed areas of grades II to III). Analysis of these tumors reveals that Cx43 behavior in gliomas is not as simple as previously reported 11, 12, 13. If our results tend to show a global diminished expression of Cx43 in glioblastomas (grade IV), they modulate some conclusions that have been presented so far. In particular, our results do not confirm the difference in Cx43 expression which was observed between grades II and III 11, 13. More generally, our study emphasizes the importance of realizing *in situ* approaches in the brain tumor context. Indeed, because of the high heterogeneity of such tumors, any global molecular analysis (such as Western blotting analysis) could be misleading and would not have revealed the *in situ* heterogeneous and aberrant localization of Cx43 as a frequent phenomenon which is probably associated with localized abnormal cell behavior.

## Materials and Methods

### Materials

Tissues from 85 adult patients harboring gliomas were collected during surgery at the Service of Neurosurgery (Poitiers University Hospital, France), with signed informed consent of patients and approval of local ethics committee. Tumor diagnosis and grading were established according to the WHO criteria 16 and were revised by two expert pathologists. This study included formalin‐fixed paraffin‐embedded gliomas: 24 grade II, 18 grade III, 37 grade IV, and 6 grade II in evolution to grade III (Table 1). Additional frozen material, snap frozen immediately after operation and stored at −80°C, was also available for 14 of these tumors.

**Table 1:** List of human glioma samples used for the study as entire slices (A) or as tissue microarrays (B)

Grade	Classification (number of patients)	Total of patients/grade	Gender	Median age (range)
A. Repartition of the human gliomas studied on entire slices				
II	Astrocytoma	5	3♂/2♀	34 (16–34)
III	Anaplastic astrocytoma 1	6	4♂/2♀	51 (41–58)
	Anaplastic oligoastrocytoma 5			
IV	Glioblastoma	15	7♂/8♀	61 (40–78)

Grade	Classification (number of patients)	Total of patients/grade	Gender	Median age (range)	“Normal” tissue available (number of samples)
B. Repartition of the human gliomas studied on tissue microarrays.					
II	Astrocytoma	19	14♂/5♀	41 (16–76)	6
III	Anaplastic astrocytoma 7	12	8♂/4♀	43 (30–62)	2
	Anaplastic oligoastrocytoma 5				
IV	Glioblastoma	22	11♂/11♀	66 (39–78)	2
II–III	Astrocytoma with anaplastic area	6	4♂/2♀	45.5 (34–67)	1

### Tissue microarray

Original slides from the 85 samples were reviewed by a pathologist to locate the tumor area. In 11 samples, nontumoral tissue was observed surrounding the tumor. In six samples, two distinct areas (grade II and grade III) were seen. For each case, three cores of tissue (0.6 mm diameter) were transferred from the selected areas to the recipient block. Serial sections of the tissue microarray (TMA) block were cut 5‐*μ*m thick and stained with hematoxylin‐eosin to verify that cores represented the selected areas.

### Immunohistochemistry

Immunostaining was performed on TMA sections and on whole mounted sections from 59 and 26 samples, respectively (Table 1). Five‐*μ*m tissue sections were placed on charged slides, baked at 60°C overnight, then deparaffinized, rehydrated, and heat pretreated for antigen retrieval with 10 mmol/L sodium citrate buffer (pH 6.0). Immunohistochemistry was performed overnight using primary antibodies against Cx43 (monoclonal mouse anti‐Cx43 antibody designed against amino acids 250–272, diluted at 1:200, Transduction laboratories, Lexington, Kentucky, USA), Glial Fibrillary Acidic Protein (polyclonal rabbit anti‐GFAP, 1:200, DakoCytomation, Trappes, France), a proliferation marker (polyclonal rabbit anti‐Ki67, 1:100, SantaCruz Biotechnology, Heidelberg, Germany), and a leukocyte marker (monoclonal mouse anti‐CD45, 1:200, DakoCytomation).

Two different staining procedures were performed depending on the following microscope observations. The first one used the ChemMate Detection kit (DakoCytomation), based on an indirect biotin‐avidin system with a universal biotinylated immunoglobulin secondary antibody, diaminobenzidin (DAB) substrate, and hematoxylin counterstain. The second protocol consisted on 45‐min incubation at room temperature with specific fluorophore‐coupled secondary antibodies: goat anti‐mouse IgG (1:400, DakoCytomation) or goat anti‐rabbit (1:400, DakoCytomation). Nuclei were stained (TO‐PRO^®^‐3; 1:10,000; InVitrogen, Courtaboeuf, France). Slides were finally protected from photobleaching by mounting media (Vectashield). Negative control slides were obtained after either omitting the primary antibody or incubating with an irrelevant antibody (mouse monoclonal immunoglobulin, IgG). Controls were run in same conditions and same IgG concentration as used for the respective primary antibodies. DAB‐stained slides were observed using Zeiss Axioskop microscope and acquired by Kappa Image‐Base software. Immunofluorescence staining was studied by an Olympus FV1000 confocal microscope and acquisitions were done with Fluoview FV10‐ASW 1.6 software. NIH Image J software (http://rsb.info.nih.gov/ij/) was used to quantify stained cells and data were analyzed with Prism software. Positive cells were expressed as a percentage of total cells in the tumor core. Immunolabeling has been performed in triplicate for each sample.

### Protein isolation and Western blot analysis

Proteins were isolated from 14 frozen tumor tissues using radioimmune precipitation lysis buffer (50 mmol/L Tris‐HCl; pH 8.0; 1% IGEPAL) supplemented with Mini Complete protease inhibitors (Roche Applied Science, Meylan, France) and phosphatase inhibitors (Sigma, L’Isle d’Abeau, France). Protein concentrations were determined using DC Protein assay quantification kit (Bio‐Rad, Marnes la Coquette, France). Protein samples were boiled for 2 min in sodium dodecyl sulfate (SDS) sample buffer (pH 8.0) and separated on a polyacrylamide gel in parallel with molecular weight markers (Bio‐Rad). Subsequently, electrophoresed proteins were transferred onto a nitrocellulose membrane (Bio‐Rad) at 100 V for 1 h. The blots were then blocked with 5% dry milk in phosphate‐buffered saline (PBS; pH 7.4; with 1% Tween 20) for 1 h and incubated with monoclonal Cx43 antibody at 4°C overnight. After rinsing with PBS, blots were incubated in horseradish peroxidase‐tagged secondary antibody (DakoCytomation) for 1 h at room temperature, followed by incubation in SuperSignal chemiluminescent substrate (Pierce). To ensure equal loading of protein samples, blots were stripped of their Cx43 antibody and reprobed for glyceraldehyde‐3‐phosphate dehydrogenase (GAPDH; 1:10,000; Clinisciences, Nanterre, France). Cx43‐transfected rat C6 glioma cells (clone C6‐13) which were used as a positive control for Cx43 expression are a kind gift from Dr C. Naus (University of British Columbia, Vancouver, Canada).

## Results

### Expression and localization of Cx43 vary within a same tumor

**Figure 1:**
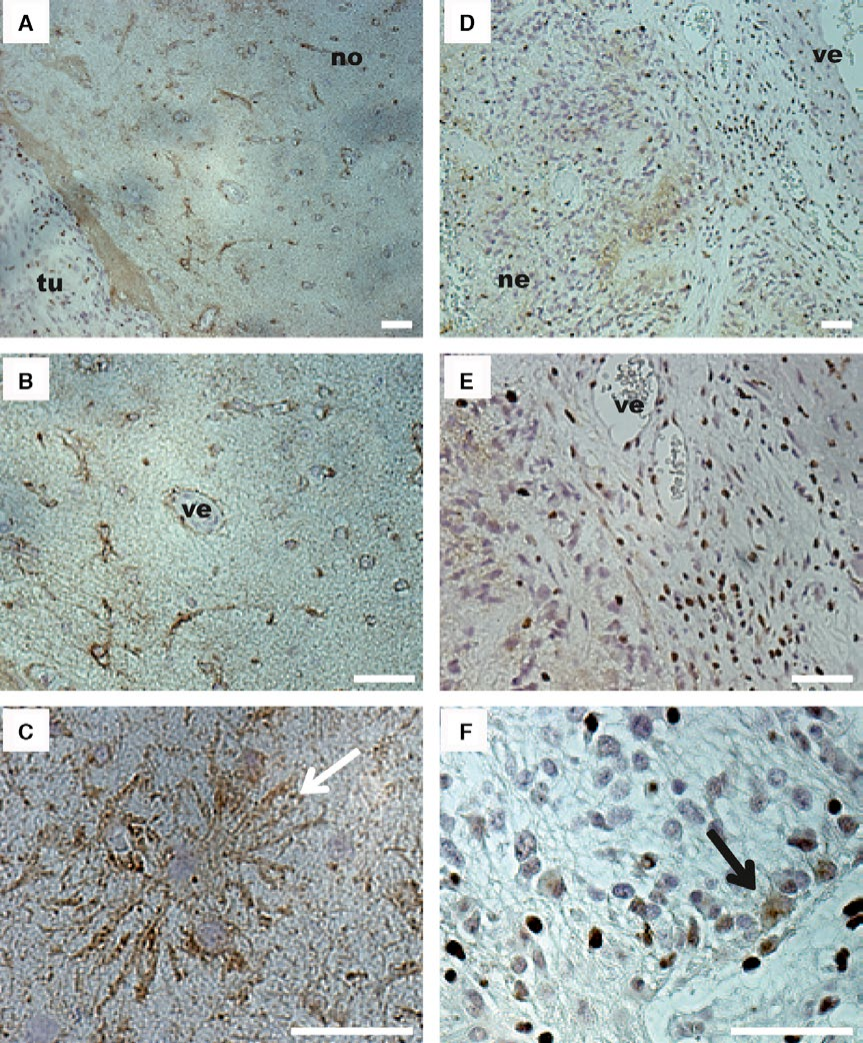
Cx43 expression observed by immunohistochemistry in pathological and surrounding areas of a grade IV glioma. (A) The surrounding nontumor area (no) exhibited a lower cell density than the tumor area (tu). Surrounding nontumor area (B–C). (B) In this part of the tissue, Cx43 staining (brown) was observed around astrocytes and at the periphery of vessels (ve). (C) At higher magnification, Cx43 staining appeared at the membrane of astrocytes (white arrow) and a more diffuse staining corresponding to the fibrillary background was observed. Tumor area (D–F). (D) Within the tumor, the tissue was disorganized and exhibited high cell density, vascular proliferation (ve) with a thick endothelium, necrotic zones (ne), and a diffuse staining for Cx43 in some parts of the tumor (brown labeling). (E) Surrounding the newly formed blood vessel (ve), Cx43 was not detected. (F) Some cells in the tumor area exhibited Cx43 in the cytoplasm (black arrow). Bar: 50 *μ*m.

First, slices of 26 entire tumor samples were studied (Table 1A). This global approach permitted to identify different zones within the tumors. Some of them presented different cell densities, whereas necrotic and neovascularization areas could be detected specifically within grade IV gliomas. Beyond the pathological tumor area, it was possible to identify a surrounding nontumoral area where Cx43 was mostly observed at the periphery of astrocytes and vessels (Fig. 1A and B). In this area, a weak Cx43 labeling was seen in the fibrillary background corresponding to astrocytic extensions (Fig. 1A and B). At higher magnification, Cx43 was detected at the plasma membrane of astrocytes (Fig. 1C). In the tumor area, necrosis and neovascularization zones endow the tissue with high heterogeneity. This heterogeneity was increased by variations in Cx43 expression inside the tumor area (Fig. 1D). For instance, in some parts of the tumor area, a fibrillary background was positive for Cx43 (Fig. 1D and E) or a cytoplasmic accumulation was seen (Fig. 1F). In other parts, no Cx43 labeling was observed in cytoplasms or in plasma membranes (Fig. 1E and F).

### Despite relative inverted correlation between tumor grade and Cx43 expression, Cx43 expression was heterogeneous within a same grade

**Figure 2:**
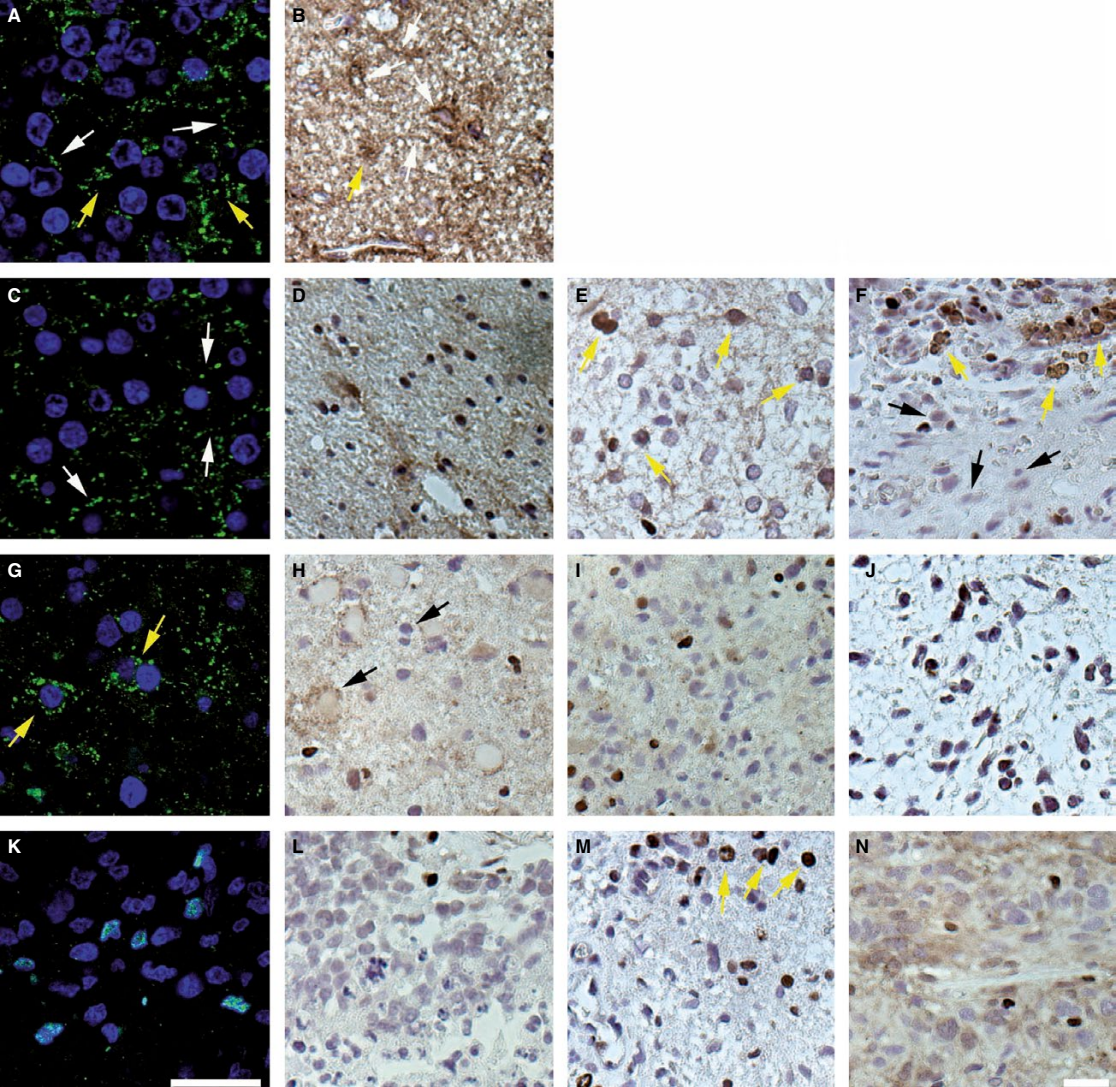
Cx43 expression in grades II–IV gliomas on tissue microarray. (A, C, G, K) Immunofluorescence for Cx43 observed by confocal microscopy (nuclei of cells were stained in blue). (B, D–F, H–J, L–N) Immunohistochemistry for Cx43. (A, B) Histologically normal tissue exhibited strong Cx43 expression at the cell membrane (white arrows) and in the cytoplasm (yellow arrows) of some cells. (C) Grade II astrocytoma. (D–F) Grade II fibrillary astrocytomas. In these four representative samples of grade II gliomas, Cx43 was expressed at the membrane (C; white arrows), in the fibrillary background (D), or in the cytoplasm (yellow arrows) of some cells (E, F). It is interesting to note that some other cells were negative (black arrows) for Cx43 (F). (G) Grade III anaplastic astrocytomas exhibited a cytosolic staining for Cx43 (yellow arrows). (H) Some oligodendrocytes were surrounded by a Cx43 staining (black arrows) possibly due to the fibrillary background of astrocytes in an anaplastic oligodendrocytoma. (I, J) In grade III anaplastic astrocytomas, Cx43 expression was limited to a diffuse brown staining (I) or was absent (J). (K–M) Grade IV glioblastoma. In the highest glioma grade, numerous cells did not exhibit any Cx43 staining (K, L). Some Cx43 staining was observed in the cytoplasm of isolated cells (M, top, yellow arrows). (N) Grade IV glioma. In this sample, the fibrillary background was stained. Bar: 30 *μ*m.

For studying the correlation existing between Cx43 expression and glioma grade, a TMA including a wide range of samples (22 grade IV, 12 grade III, 19 grade II, and 6 grade II associated with newly anaplastic areas) was used (Table 1B). In this TMA, areas surrounding the tumors were also included in which Cx43 was mostly detected at the plasma membrane of the cells, in the fibrillary background and less within the cytoplasm (Fig. 2A and B).

In grade II gliomas, the pattern of Cx43 expression was complex. It looked normal in several cases in which Cx43 was detected at the plasma membrane of some cells when investigated by confocal microscopy (Fig. 2C) or as a stained fibrillary background (Fig. 2D). However, in the majority of cases, Cx43 was observed aberrantly in the cytoplasm of the cells (Fig. 2E and F) and was even not detected in some of them (Fig. 2F). Concerning grade III gliomas, two kinds of pictures were observed depending on the tumor type which was considered, astrocytomas or oligodendrogliomas (Fig. 2G–J). In oligodendrocytic tumors, the fibrillary background was labeled and a Cx43 signal was detected also at the periphery of oligodendrocytes in every sample (Fig. 2H). In astrocytic tumors, Cx43 was not detected or only in the cytoplasm of a few cells (Fig. 2G, I, and J). In grade IV gliomas, Cx43 was not detected in most of tumor cells (Fig. 2K–M) even if some samples exhibited either a few cells with a cytoplasmic accumulation or a stained fibrillary background (Fig. 2M and N).

**Figure 3:**
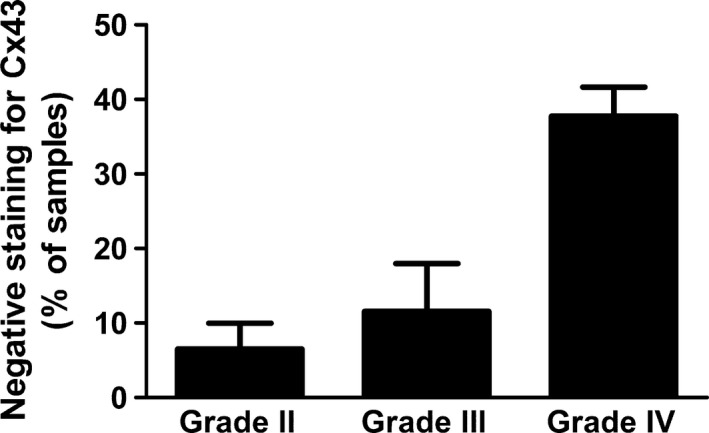
Proportion of glioma samples that did not express Cx43. Within each grade, samples were available in triplicate (See Table 1B; 19 grade II, 12 grade III, and 22 grade IV). Samples that did not exhibit any detectable staining for Cx43 in three independent experiments on serial slides were considered as Cx43 negative. On the other hand, cells were considered as Cx43‐positive cells when they exhibited Cx43 staining independently of its localization (plasma membrane or cytoplasm). Following this methodology, 6.5% of grade II tumor samples did not exhibit any staining for Cx43, versus 11.6% for grade III tumors and 37.8% for grade IV tumors.

When we tried to analyze more precisely Cx43 expression in the TMA samples (Table 1B; 19 grade II; 12 grade III; 22 grade IV), we were confronted with tumor tissue disorganization and occasional presence of a fibrillar background. In particular, such situations prevented to discriminate cells exhibiting only a cytoplasmic localization from those exhibiting both a cytoplasmic and a plasma membrane staining. Consequently, because of this difficulty, we chose to discriminate samples presenting a total lack of Cx43 from those exhibiting Cx43 staining whatever it was (cytoplasmic and/or in plasma membrane). According to this approach, we could estimate that Cx43 was not detected in 6.5% of grade II, 11.6% of grade III, and 37.8% of grade IV samples (Fig. 3). As these results were obtained from TMA, we cannot exclude completely that the small area of TMA piece (0.6 mm diameter) that was studied may not reflect all other parts of the same tumor in terms of Cx43 staining. However, this risk is limited by three considerations. First, TMA piece represents a typical part of the tumor which reflects its grade and was chosen by a pathologist. Second, heterogeneity is so high in gliomas (and even more in glioblastomas) that any heterogeneity of Cx43 staining would be detected in a 0.6‐mm‐diameter piece. And third, three cores of tissues per tumor were taken and lack of Cx43 expression was conclusive when Cx43 immunostaining was negative for the three cores of the same tumor. Because of these considerations, we estimate that Cx43 staining in TMA pieces is representative of *in situ* Cx43 expression in whole tumor.

**Figure 4:**
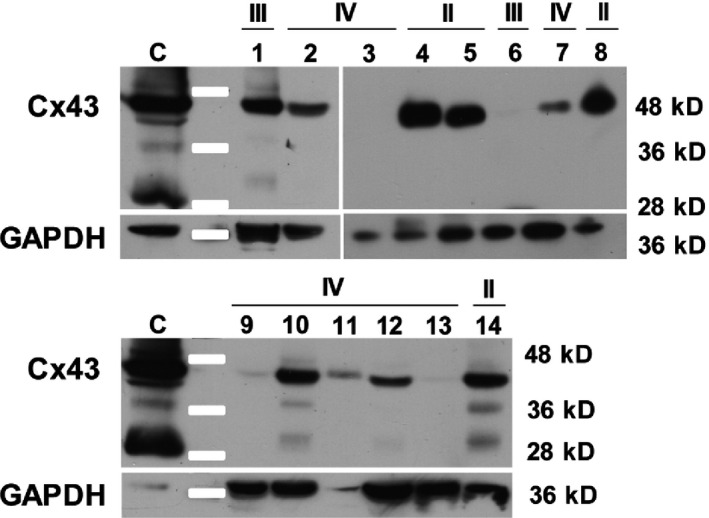
Western blot showing the variability in Cx43 expression in human gliomas. Within grade II tumors (4, 5, 8, 14), Cx43 was detected in every sample at the expected size (43 kD). Within grade III tumors (1, 6), Cx43 was shown in only one of the two samples. In grade IV tumors (2, 3, 7, 9–13), two samples out of eight did not express Cx43 at a detectable level. (C) Rat C6 glioma cell line expressing a high level of Cx43, used as a positive control. In some samples (1, 10, 14), Cx43 antibody allowed the detection of another band (25 kDa) that was also observed in the control. Moreover, in some of these samples (10, 14), a third band (35 kDa) was clearly seen as in the control (C).

As some tumor samples were kept frozen, we could study their global Cx43 expression level by Western blotting. This approach, which was realized by using 4 grade II, 2 grade III, and 8 grade IV gliomas (Fig. 4), gave heterogeneous results. For instance, Cx43 was detected as a normal 43 kDa band in all kinds of grades, but with differences depending on the samples (grade II: 4/4; grade III: 1/2; and grade IV: 6/8, but with variable intensity). No Cx43 signal was detected for some of the highest grades (grade III: 1/2; grade IV: 2/8). Two additional Cx43 bands (35 and 25 kDa) were observed in some samples exhibiting the major 43 kDa band. Interestingly, the presence of these bands was not correlated with tumor grade as it was detected in one sample of each grade (grades II–IV) and also in the positive control (rat C6 glioma cell line transfected with Cx43) 9. At least one of them, the 25 kDa band, was previously described, but not defined as a degradation product of Cx43 17.

For some patients, both small frozen and large formalin‐fixed paraffin‐embedded pieces were available from the same glioma. It was then possible to compare, for the same tumor, Cx43 expression patterns by Western blotting (from fixed tissue) and immunohistochemistry (from frozen pieces). When such comparison was possible, Cx43 level of expression occasionally appeared different by considering results obtained either by immunohistochemistry or Western blotting. For instance, for a grade IV glioma, no Cx43 signal was detected by Western blotting (Fig. 4, sample 13), whereas the embedded sample exhibited a strong Cx43 labeling within the area surrounding the tumor (data not shown).

### Cx43 nuclear staining was not characteristic of glioma cells, but might be correlated with leukocyte infiltration within the tumors

**Figure 5:**
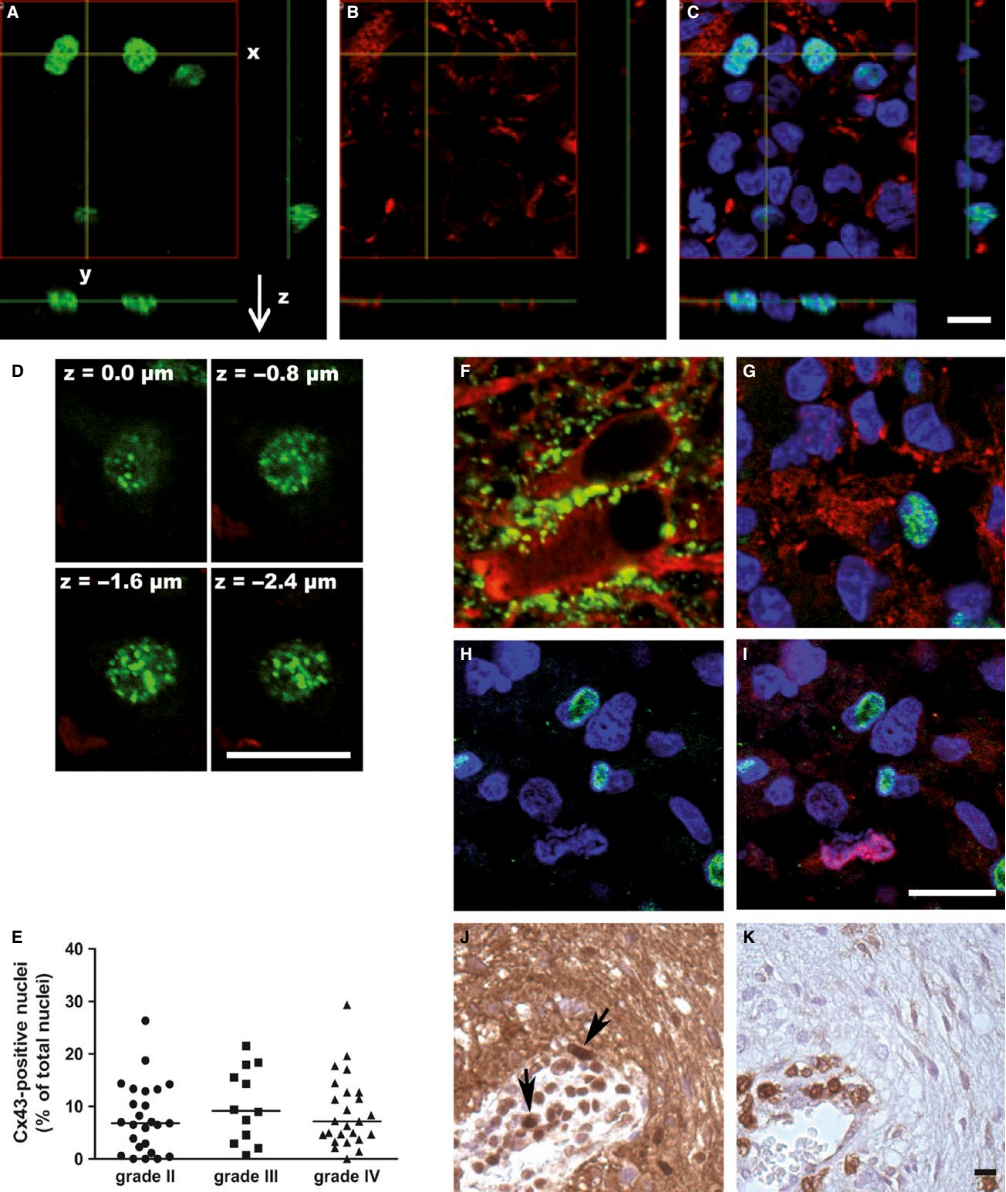
Presence of a Cx43 nuclear staining in the core of the tumors. (A, B, C) Confocal analysis of the Cx43 staining in 3D (x‐, y‐, and z‐axis). A Cx43 signal (green) was detected in the nucleus (blue) of several cells (A, C). This staining seemed to be detected in cells that did not express a specific astrocyte protein, GFAP (in red; B, C). (D) Confocal analysis of the Cx43 nuclear staining in the nucleus thickness following the z‐axis. The Cx43 signal was shown in successive focal plans within the nucleus. (E) Quantification of the Cx43‐stained nuclei in the different tumor grades (II to IV). No statistically significant difference was shown between the three groups. (F–K) Endeavor of identification of the cell type exhibiting Cx43‐stained nuclei. In the surrounding nontumor area (F), a characteristic Cx43 staining (in green) was shown at the cell–cell contact areas between astrocytes (GFAP, in red). In the core of the tumor (G), a cell exhibiting a Cx43‐stained nucleus (green and blue) appeared GFAP negative, on the contrary of surrounding cells (blue nuclei) that were GFAP positive (red) and did not show Cx43 expression. In the core of the tumor (H, I), costaining was performed for the proliferation nuclear‐marker Ki67 and Cx43. The nucleus of proliferating cells (in red; I) did not colocalize with the Cx43 nuclear staining (in green; H, I). As an accumulation of Cx43 nuclear staining was seen surrounding the newly formed vessels (J; in brown; black arrows), histochemistry assays were made on serial slides for the leukocyte marker CD45 (K; in brown). The areas of high Cx43 nuclear staining appeared correlated with the CD45‐positive cells, especially close to the endothelium. Bar: 50 *μ*m.

A Cx43 nuclear signal was detected in a large amount of samples (Figs. 1, 2). As shown by confocal analysis, this signal was localized inside the nucleus of the cells (Fig. 5A–D), but not related to tumor grade (Fig. 5E). In order to identify which cell type exhibited such a signal, we checked whether those cells were from an astrocytic lineage by testing the expression of the glial fibrillary acidic protein (GFAP). As expected in normal tissue, colabeling of Cx43 and GFAP revealed that Cx43 was detected in the plasma membrane of astrocytes (Fig. 5F). In the tumor area, even if GFAP labeling was weaker and diffused, cells exhibiting Cx43 nuclear staining were not GFAP positive (Fig. 5A–C and 5G). As one of the characteristics of gliomas is their mitotic index, colabeling of Cx43 and Ki67 was done in order to see whether the Cx43 nuclear signal was associated with cell proliferation. Confocal analysis of Cx43 and Ki67 labeling indicated that cells exhibiting a nuclear signal are different from Ki67‐positive cells (Fig. 5H, I). Finally, as a higher number of cells with a Cx43 nuclear signal was close to newly formed vascular endothelium and leukocyte infiltrations, we checked for the presence of leukocytes exhibiting such a signal. By using anti‐CD45 antibody to identify leukocytes, we observed in serial slices that both nuclear Cx43‐positive cells and CD45‐positive cells were detected in blood vessels or close to the vascular endothelium (Fig. 5J and K). However, by such an approach, we could not prove definitely that CD45‐positive cells and nuclear Cx43‐positive cells are the same. The only conclusion we can draw is, as Cx43 nuclear staining was more detected in areas where leukocytes are present, it suggests that such staining might be associated with leukocyte infiltrations in brain tumors 18.

## Discussion

The aim of our study was to define more precisely the link existing between Cx43 expression and human glioma grading. This study was based on previous attempts made by others and showing an inverse relationship between Cx43 expression and glioma progression: higher is grading and lower is connexin expression 11, 12, 13, 14. As human gliomas are known to exhibit high tissue heterogeneity, it was important to consider the *in situ* behavior of Cx43 in such a heterogeneous cellular context by using *in situ* immunohistochemistry. This approach appeared to us more informative instead of analyzing global Cx43 expression by Western blotting. Indeed, if a Western analysis may suggest that Cx43 level decreases with grading, it does not inform in which cell type it occurs or in which area of the tumor. Therefore, the objective of our study was to check whether any aberrant expression of Cx43 could be associated with either a particular grade of glioma progression, any characteristics of the tumor cells, or any distinct tissue architecture. And as most of studies so far were carried out on a small number of cases, we performed our analysis on a higher number of samples illustrating each diagnosed grade of glioma progression. Conciliating high number of cases and *in situ* analysis of Cx43 expression on selected areas of the tumor was possible by using the TMA technique in comparison with more classical and global approaches such as Western analysis.

To do so, we analyzed by immunohistochemistry and Western blotting approaches a large number of glioma samples for Cx43 expression: 79 gliomas (grading from II to IV) and 6 grade II samples progressing to grade III. The immunohistochemistry approach revealed that Cx43 is heterogeneously expressed in grade II samples. Even at such a low grade, Cx43 was found aberrantly present in the cytoplasm of tumor cells or not detected. These characteristics, which were also present in grade III astrocytomas, prevent differentiating grading evolution from grade II to III by Cx43 expression. However, and this is an interesting observation, the oligodendrocytic grade III samples were characterized by heavy Cx43 labeling. Therefore, the level of Cx43 expression might help distinguishing astrocytic and oligodendrocytic grade III subtypes. Finally, glioblastomas (grade IV) were macroscopically characterized by marked tissue heterogeneity (necrotic areas and neovascularization) which was associated with high *in situ* heterogeneity of Cx43 expression inside the tumor. Indeed, in most of these glioblastomas, Cx43 labeling was not detected but still present in some areas either as an aberrant cytoplasmic staining or as normal fibrillary background. Thus, from grade II to grade IV (and through grade III astrocytomas), a dual aberration of Cx43 expression was constantly observed as either a lack of detection or a cytoplasmic accumulation in the cells. From our study, it seems that among these two major aberrant expressions of Cx43, only lack of expression was increased with tumor grading.

Because of heterogeneity of Cx43 expression in the samples, this decreased *in situ* detection with grading was not so obvious by Western analysis. In grade IV samples, the heterogeneity of Cx43 expression was so high that we did not observe any correlation between Western analysis and immunohistochemistry for the same sample. Such an observation suggests *in situ* approaches are more informative than global ones for studying the involvement of particular proteins, such as Cx43, in glioma progression.

From such an analysis, if we confirm that Cx43 expression is globally diminished in the higher grades of gliomas, our work presents important differences with previous studies. For instance, we have shown that Cx43 expression is maintained in nearly 90% of grade II samples instead of 57% in a previous study 13. We also observed that cells expressing Cx43 can still be detected in the majority of glioblastomas (62% of cases). This observation can be compared with another study which was performed on 24 astrocytic tumors (8 of each grade) and exhibited percentages of Cx43 expression very close to what we obtained 19. Despite this similarity, our results are different from this work when considering oligodendrocytic tumors. In such tumors, we observed strong localization of Cx43 at the periphery of cells presenting an oligodendrocyte phenotype. As oligodendrocytes are known to express another connexin, Cx32, this presence of Cx43 is intriguing and would need more investigation. However, it is also known that aberrant Cx43 expression occurs in cancer cells which do not normally express it. For instance, Cx43 is detected in human hepatocellular carcinomas contrary to normal hepatocytes which express Cx32 and Cx26 20.

Another important difference between our results and previous studies concerns astrocytomas of grades II and III. Contrary to what was postulated 11, 13, we could not find any significant difference in Cx43 expression between these two grades. Thus, our work suggests that Cx43 expression or localization cannot be used as a marker for astrocytic tumor grading, even for distinguishing grades II and III. Moreover, it does not help for a better characterization of glioblastomas. The fact that Cx43 is still detected in high‐grade gliomas (grades III and IV) may be in favor of the presence of possibly invasive cells as glioblastoma are known to be very invasive and Cx43 has been involved *in vitro* in glioma migration and invasion 21, 22. Future *in situ* studies will determine whether Cx43 is related to *in situ* glioma invasion even for low‐grade tumors. This task may not be easy as it needs identifying isolated and invasive Cx43‐positive cancer cells infiltrating a normal Cx43‐rich astrocyte background. Apart its involvement in cell migration, Cx43 has been described as controlling negatively proliferation of glioma cell lines 9, 10. Such results might explain the increased *in situ* ratio of Cx43‐negative cells correlated with grading. Indeed, it is possible that those Cx43‐negative cells, as variants of a genetically heterogenous tumor cell population, would proliferate more than Cx43‐positive cells and become the most frequent cells in some areas of glioblastomas.

Another original part of our work is the detection of a Cx43 nuclear signal in all grades and, to some extent, at the periphery of the tumor core. Our attempts to detect Cx43 by Western blotting in nuclear extracts from frozen tissues could not be done because of the limitation of available tissues 23. The human glioma pieces were too small to make such a study. Even if it is intriguing, nuclear localization of Cx43 was also observed in human colorectal tumors. Interestingly, such a localization did not appear related to tumor grade but with longer survival of the patients 24. *In vitro*, such a signal has been also previously detected in nucleus of rat liver epithelial cells transfected with oncogenes 25 and in human glioma cell lines transfected with Cx43 10. Transfection of Cx43 (or its carboxy‐terminal part) in HeLa cells, HEK 293 cells, and cardiomyocytes also leads to the detection of a Cx43 nuclear signal associated with a decreased cell proliferation 26, 27.

The fact that the cytoplasmic carboxy‐terminal part of Cx43 (15–20 kDa) was detected in the nucleus of transfected Hela cells makes possible the presence of a Cx43 nuclear signal in gliomas. Indeed, supplementary bands from 25 kDa to 35 kDa were detected by Western blotting in gliomas whatever their grade (Fig. 4). As the monoclonal antibody we used recognizes an epitope which is located in the carboxy‐terminal part of the Cx43, it is possible that the Cx43 nuclear signal detected is the consequence of the presence of this cytoplasmic portion in the nucleus of those tumors. Our attempts to identify cell types expressing a nuclear Cx43 signal revealed that it was not present in cells harboring characteristics of glioma cells such as GFAP (marker of astrocytic cells) or Ki67 (marker of proliferative cells). A possible correlation with CD133‐positive stem cells could not be either demonstrated (data not shown). On the other hand, this signal appeared to be detected in relation to the presence of CD45‐positive cells (leukocytes). Further investigations will be necessary to demonstrate its significance. However, on a fundamental point of view, this work is one of the first ones to report a Cx43 nuclear signal *in situ* as previous studies only detected it *in vitro* 10, 26, 27, 28.

In conclusion, as Cx43 was described *in vitro* to be involved in proliferation and invasion of glioma cells, we checked *in situ* any evolution of its expression during glioma progression. In order to prevent inconsistent observations due to high heterogeneity of these tumors, we avoided studying small series of samples, but dealed with a high number of samples (85 samples) covering progression of adult gliomas from grade II to IV. To harmonize our observations, we selected parts of the tumors by using TMA technique and privileged *in situ* approach to take into account the tissue heterogeneity. When our *in situ* approach was compared to global analysis of Cx43 expression such as Western blotting, we observed that the results could be misleading by following the global approach. Because of these misleading interpretations, an important conclusion of this study is the necessity to work on a wide range of glioma samples by using *in situ* approach whatever the protein of interest is. Among the different possible *in situ* approaches, we estimate that studying subcellular localization of the final product (the protein) brings more definitive informations than studying the presence of intermediate products such as mRNA (*in situ* hybridization). This is particularly true for glioblastomas in which Cx43 mRNA may be present but not the protein 14.

Despite these considerations, Cx43 does not appear being a stringent biomarker for diagnosis and estimation of glioma grading except for grade III oligodendrocytic gliomas which express more Cx43 than grade III astrocytic gliomas. Even the nuclear detection of a Cx43 signal was not related with glioma grading but possibly to leukocyte infiltrations. As previous studies did, our work shows that Cx43 expression globally decreased with grading. But as we observed, this decrease is accompanied by a high heterogeneity of Cx43 expression (lack of detection, cytoplasmic expression, and fibrillary background) starting from grade II gliomas. This *in situ* heterogenous expression of Cx43 may be related to some studies realized *in vitro* showing its involvement in some aspects of the biology of glioma cells (proliferation and invasion). However, such a relation is not firmly established yet and needs further *in situ* analyses implicating other key proteins involved in those phenomenons in relation with Cx43 expression. This kind of analysis is crucial as it may bring some insights on Cx43 as a prognosis factor and could reveal new therapeutic targets especially against the invasion of brain parenchyma by glioma cells.

## Conflict of Interest

None declared.
